# Supplementary material for: Integrated analysis of the aqueous humor microbiome and lens capsule transcriptome in high myopia cataract: a pilot study
Source: Front Med (Lausanne). 2026 Jun 16;13:1845205. doi: 10.3389/fmed.2026.1845205 (PMC13314463; doi:10.3389/fmed.2026.1845205)
Supplement: Supplementary file 3 [file Data_Sheet_1.zip › 3.Beta_Diversity/PCoA/C372089/PCoA_binary_jaccard_2D_nolabel.pdf]

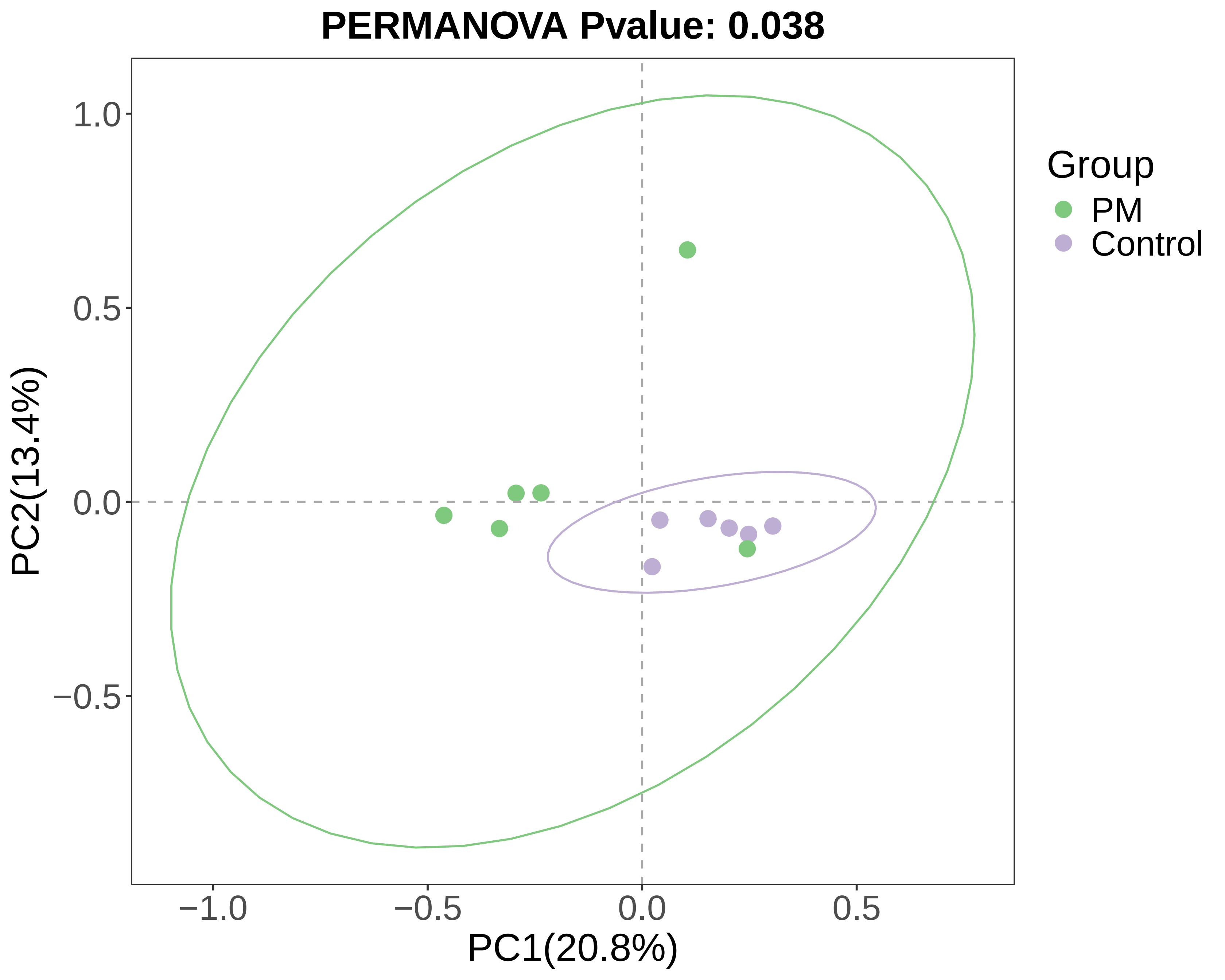
PERMANOVA Pvalue: 0.038
1.0
0.5
0.0
−0.5
−1.0
−0.5
0.0
0.5
PC1(20.8%)
PC2(13.4%)
Group
PM
Control
